# Factors Associated With Hip Fracture Length of Stay Among Older Adults in a Community Hospital Setting

**DOI:** 10.5435/JAAOSGlobal-D-22-00195

**Published:** 2023-05-02

**Authors:** Eric Schweller, James Mueller, Olga J. Santiago Rivera, Sergio J. Villegas, Joseph Walkiewicz

**Affiliations:** From the Garden City Hospital, Orthopedic Surgery Residency Program, Garden City, MI (Dr. Schweller, Dr. Mueller, and Dr. Walkiewicz); the Graduate Medical Education , Garden City Hospital, Garden City, MI (Dr. Santiago Rivera); and the Michigan State University College of Osteopathic Medicine, East Lansing, MI (Dr. Santiago Rivera and Dr. Villegas).

## Abstract

Hip fractures in geriatric populations constitute a heavy burden on the healthcare system. The study goal was to identify patient, hospital, and surgical factors associated with hospital length of stay (LOS) for geriatric patients with hip fractures requiring surgical intervention in a community hospital setting.

**Methods:**

This was a cross-sectional retrospective chart review of geriatric hip fractures that underwent surgical fixation at a community hospital between 2017 and 2019. The scope of the surgeries was limited to cephalomedullary device fixation or hemiarthroplasty hip fracture surgeries. Sliding hip screw or total hip arthroplasty procedures and patients who died during the index hospitalization were excluded. Median tests were conducted to examine differences between the groups. Unadjusted and adjusted truncated negative binomial regression models were used to examine the factors associated with LOS.

**Results:**

Bivariate analyses revealed results that the factors associated with a longer LOS were preoperative anemia (*P* = 0.029), blood transfusion (*P* = 0.022), and the number of days between admission and surgery (*P* = 0.001). The adjusted regression model results suggested that older patients, patients who underwent surgery more than one day after admission, current smokers, malnourished patients, patients with sepsis, and patients with a history of a thromboembolic event had statistically significant (*P* < 0.05) longer LOS. However, patients who live in institutions (nursing homes or assisted living) had a shorter LOS than those who live at home alone or with family (*P* < 0.05).

**Discussion:**

Older adult patients who underwent surgery with a cephalomedullary device or hip hemiarthroplasty for a hip fracture and had preoperative anemia, postoperative blood transfusions, and increased days between admission and surgery had a longer LOS. Additional factors positively associated with a longer LOS included current smokers, malnourishment, admission with sepsis, and patients with a history of a thromboembolic event. Of interest, institutionalized patients had a shorter LOS than those living at home alone or with family.

Hip fractures in adults aged 65 years or older (geriatric hip fractures) constitute a heavy burden on the healthcare system and are associated with increased utilization of surgery, hospital resources, and rehabilitation services. In the United States, the incidence of hip fractures among adults aged 65 years or older is approximately 950 of every 100,000.^1^ Epidemiologic studies suggest this incidence to be stabilizing despite the aging population but continues to be a high burden on society.^1^ Previous reports in Europe and the United States have both shown a trend toward a decrease in inpatient length of stay (LOS) after surgical interventions treating geriatric hip fractures.^2,3^ However, in clinical practice, some patients require longer inpatient postoperative recovery, and in the United States specifically, increased LOS has been shown to be correlated with increased postoperative mortality.^2^

The American Academy of Orthopaedic Surgeons has well-established guidelines on how to manage these patients in the hospital and how to surgically treat these fractures.^4^ However, there continues to be a wealth of newly published peer-reviewed journal articles studying geriatric hip fractures. The volume of articles is likely an indicator of unsolved research questions and gaps in the literature, as these patients typically have multiple medical comorbidities and managing them in the hospital can be difficult. The medical comorbidities often include respiratory syndromes, cardiac conditions, dementia, renal impairment, and diabetes.

Identifying patients who require a longer LOS can be an important metric useful for stratifying patients based on their risk of complications after hip fracture surgery. Multiple studies have shown links between longer LOS with higher postsurgical mortality after hip fracture surgeries in the geriatric population.^1,2^ However, there has been a limited number of publications focused on the factors that are associated with increased hospital LOS. Furthermore, most of the literature comes from larger trauma centers and/or academic centers. It remains to be seen whether the results seen in these larger centers are seen in smaller community hospitals. The main aim of this study is to identify patient, hospital, and surgical factors associated with hospitalization LOS of older adults with hip fracture surgeries in a community hospital setting. In addition, we hope to offer suggestions on how to optimize these factors at a community hospital level to reduce the LOS. We hypothesize that the results from our community hospital will be similar to the results of other studies that found preoperative anemia, receiving transfusions, smoking, body mass index (BMI), delay in surgery from time of admission, and coming from home or living with family will be factors that increase the LOS.

## Methods

This was a cross-sectional retrospective chart review study. The study protocol was reviewed and approved by the cognizant institutional review board for the protection of human subjects in research.

The study population consisted of adults aged 55 years or older who were admitted to a 176-bed community-based hospital in the Detroit, MI, area with no trauma level designation from January 1, 2017, to December 31, 2019. Patients were admitted primarily for a hip fracture (femoral neck or intertrochanteric femur) and were subsequently treated with surgical management (*ICD-10*: 470). The patients were treated by board-certified orthopaedic surgeons: a general orthopaedic surgeon, two adult reconstruction fellowship-trained orthopaedic surgeons, and a sports medicine fellowship-trained orthopaedic surgeon. We made the decision to include patients aged 55 years and up to increase our study population and to capture as many patients as possible who were treated with either a hip hemiarthroplasty or a cephalomedullary device. Exclusion criteria consisted of patients who did not have a hip fracture at the time of admission, cases with hip fracture surgeries that were not cephalomedullary device fixation or hemiarthroplasty, and patients who died during the index hospitalization. We excluded sliding hip screws because we did not have enough cases using that implant for proper analysis. In total, 166 medical records were reviewed. From these records, nine patients were excluded: six died during the hospitalization, and three had other types of surgery. The resulting study sample consists of 157 patients.

The key response variable in this study was in-hospital LOS for the hip fracture index hospitalization. It was defined as the number of days from the admission date to the discharge date. The discharge date was defined as the date when the patient was discharged to home or transferred to another institution (eg, nursing home, long-term acute care facility, and inpatient rehab).

The potential patient-level factor associated with hip fracture surgery LOS (HFSLOS) among older adults included sociodemographic characteristics such as age, sex, race, marital status, health insurance (ie, Medicare coverage, other health insurances, or third-party payers), and living arrangements (ie, home or living with family, other living arrangements such as a nursing home, group home, or assisted living). Health behaviors (ie, current smoking and alcohol use), health status indicators measured during the hospitalization, and comorbidities at the time of the admission were also examined.

Health status indicators measured during the hospitalization included nutritional status, body mass index (BMI), preoperative anemia status, and concurrent sepsis. Preoperative nutritional status was evaluated using albumin; values ≤3.4 mg/dL were considered malnourished. Albumin was chosen as a surrogate marker for malnutrition because it is the most well-established marker for malnutrition.^2^ Preoperative anemia was categorized based on the hemoglobin (HgB) level and was defined as Hgb <13.5 for males and <12 for females.^5^ For BMI (kg/m^2^), we used the standard weight status categories based on the World Health Organization (WHO) values: underweight (below 18.5 kg/m^2^), normal (18.5 to 24.99 kg/m^2^), overweight (25.0 to 29.99 kg/m^2^), and obese (30 kg/m^2^ and above).^6^ Patients were classified with concurrent sepsis following the systemic inflammatory response syndrome (SIRS) criteria (Bone et al., 1992).^7^ At least two of the following four criteria should be present to categorize the patient with sepsis: (1) temperature higher than 38°C or lower than 36°C; (2) heart rate (HR) greater than 90 beats/min; (3) respiratory rate (RR) above 20 breaths/min or arterial partial pressure of carbon dioxide tension (PaCO_2_) lower than 32 mm Hg; and (4) white blood cell counts less than 4 × 109/L or greater than 12 × 109/L or more than 10% bands.

For this study, we examined the association of hip fracture surgery LOS with several health conditions reported in the patient's medical history, including high blood pressure (HTN), hyperlipidemia (HLD), diabetes mellitus (any type), thyroid disorders, gastrointestinal diseases, renal dysfunction, atrial fibrillation, nonmyocardial infarction (MI) heart disease (eg, congestive heart failure and pacemaker), thromboembolic event (ie, stroke, MI, or deep vein thrombosis), lung pathology, any cancer, and mental health conditions (ie, dementia and self-reported psychological treatment).

In terms of hospitalization and surgical factors, we evaluated the association of LOS with admission date, admission ward, length of the period (in days) between admission date to surgery date, day of the week when the surgery was conducted, type of surgery, admission team, surgeon, blood transfusion(s) during hospitalization, date of the week of the patient's discharge, and to where the patient was discharged (ie, inpatient rehabilitation (IPR) facility, skilled nursing facility, home with home care, and long-term acute care). In addition, we evaluated the association with the preoperative physical status classification system of the American Society of Anesthesiologists (ASA).^8^ The preoperative rating ASA score (range 1 to 5) assesses and communicates preanesthesia medical comorbidities, from 1, representing a normal healthy patient, to 5, representing a patient not expected to survive longer than 24 hours. The ASA score was determined the day of the surgery by the anesthesiologist after evaluating the patient.

## Statistical Analyses Plan

To estimate the characteristics of the study sample, our statistical approach consisted of means, medians, frequencies, and percentages. Bivariate analyses including nonparametric equality-of-medians test were conducted to examine differences between groups (eg, sex). In subsequent analysis, the statistical approach involved unadjusted and adjusted regression models to examine factors associated with the LOS.

We performed an investigator-guided stepwise approach to determine the more parsimonious regression model. The variables included in the initial regression model were sociodemographic characteristics that have been associated with LOS (eg, age, race, and sex) and variables that were statistically significantly associated with LOS (ie, preoperative anemia, blood transfusion, and days from admission to surgery) in the bivariate analyses. We incorporated other variables into the model while testing for statistical significance and evaluating for collinearity.

In this analysis, we stressed the precision of the study estimates with a focus on 95% confidence intervals, and *P* values will be presented as an aid to interpretation. All analyses were conducted with Stata 15.1.

## Results

### Description of the Study Sample

The mean LOS was 5.36 days (range 1 to 19 days), and the median duration of stay at the hospital was 5.0 days (IQR 4-6 days) (Table 1). In our study sample, the average age of the patients was 83.9 years (SD = 7.9). Most were female (73.3%, n = 115) and White (93%, n = 145) (Table 1). Only 13.3% (n = 20) were current smokers, and 11.8% (n = 18) were current alcohol users. Almost half of the patients (47.4%, n = 74) were in a normal BMI range (18.5 to 24.99 kg/m^2^); however, 53.6% (n = 82) were malnourished, and 58.6% had preoperative anemia (n = 92) (Table 2).

**Table 1:** Distribution, Mean, and the Median of Length of Stay (Days), by Sociodemographic Characteristics (N = 157)

Characteristic	Percent (n)	Mean LOS (SD)	Median^a^ (IQR) LOS	Range LOS	*P* value^b^
Length of stay		5.36 (2.7)	5.0 (4.0.6.0)	1–19	
Age (yrs)		83.9 (7.86)		70-103	
Sex					
Female	73.3 (115)	5.1 (2.3)	5.0 (4.0.6.0)	1-15	0.092
Male	26.8 (42)	6.1 (3.5)	5.0 (4.0.7.0)	2-19	
Race					
White	93.0 (145)	5.4 (2.8)	5.0 (3.0.6.0)	1-19	0.747
African/American	7.0 (11)	5.1 (1.0)	5.0 (5.0.6.0)	3-7	
Marital status					
Married/domestic	30.5 (46)	5.3 (3.2)	5.0 (4.0.6.0)	2-19	0.277
Partnership					
Divorced	11.9 (18)	4.9 (1.5)	5.0 (4.0.6.0)	3-8	
Single	15.2 (23)	4.5 (1.7)	4.0 (3.0.6.0)	3-8	
Widowed	42.4 (64)	5.9 (2.9	5.0 (4.0.7.0)	3-15	
Insurance					
Medicare (any coverage)	80.9 (127)	5.2 (2.5)	5.0 (4.0.6.0)	1-19	0.403
Other health insurance	19.1 (30)	6.0 (3.3)	5.0 (3.0.8.0)	2-13	
Living arrangements					
Home or living with	85.3 (133)	5.4 (2.8)	5.0 (4.0.6.0)	2-19	1.000
Family					
Other living arrangements	14.7 (23)	5.0 (2.1)	5.0 (3.0.6.0)	1-10	

IQR = interquartile range, LOS = length of stay

a Median, Fisher exact test.

b Analyses were restricted to patients without missing values.

**Table 2:** Distribution, Mean, and Median of Length of Stay (Days), by Health Behavior and Health Status Indicators (N = 157)

Characteristic	Percent (n)	Mean LOS (SD)	Median^a^ (IQR) LOS	Range LOS	*P* value^b^
Smoking status					
Never	64.0 (96)	5.2 (2.6)	5.0 (3.0,6.0)	1-19	0.120
Former	22.7 (34)	5.5 (2.1)	5.0 (4.0,7.0)	2-10	
Current	13.3 (20)	6.6 (3.7)	5.5 (4.0,7.0)	3-15	
Alcohol use					
Never	85.5 (130)	5.2 (2.6)	5.0 (3.0,6.0)	1-19	0.407
Former	2.6 (4)	6.0 (2.9)	5.5 (4.0,8.0)	3-10	
Current	11.8 (18)	6.6 (3.5)	5.5 (4.0,8.0)	2-13	
Nutritional status					
Nonmalnourished	46.4 (71)	4.7 (2.0)	4.0 (3.0,6.0)	1-13	0.093
Malnourished	53.6 (82)	5.8 (3.1)	5.0 (4.0,7.0)	2-19	
Body mass index (kg/m^2^)					
Underweight (<18.5)	11.5 (18)	5.9 (3.4)	4.5 (4.0,7.0)	3-15	0.776
Normal (18.5-24.99)	47.4 (74)	5.5 (3.0)	5.0 (3.0,6.0)	2-19	
Overweight (25.0-29.99)	29.5 (46)	4.9 (1.9)	5.0 (3.0,6.0)	1-11	
Obese (≥30)	11.5 (18)	5.4 (2.2)	5.0 (4.0,7.0)	2-10	
Preoperative anemia^c^					
No	41.4 (65)	4.7 (2.5)	4.0 (3.0,6.0)	1-15	0.029
Yes	58.6 (92)	5.9 (2.7)	5.0 (4.0,7.0)	3-19	
Concurrent sepsis—SIRS criteria					
No	93.0 (146)	5.3 (2.7)	5.0 (4.0,6.0)	1-19	0.208
Yes	7.0 (11)	5.8 (2.4)	6.0 (4.0,8.0)	3-11	
Blood transfusion					
No	80.2 (126)	5.2 (2.8)	4.0 (3.0,6.0)	1-19	0.022
Yes	19.8 (31)	6.0 (2.0)	6.0 (5.0,7.0)	3-11	

IQR = interquartile range, LOS = length of stay, SIRS = systemic inflammatory response syndrome

a Median, Fisher exact test.

b Analyses were restricted to patients without missing values.

c HgB normal levels: 14-17 g/dL for men and 12-15 g/dL for women.

The most frequently reported comorbidities were HTN (66.2%, n = 104), hyperlipidemia (38.2%, n = 60), and nonmyocardial infarction heart disease (26.8%, n = 42) (Table 3). Most of the patients had an ASA score of III (66.9%, n = 105) and spent between 1 and 2 days from admission to surgery (73.3%, n = 115) (Table 4). The type of surgery was either hemiarthroplasty (57.3%, n = 90) or cephalomedullary device fixation (42.7%, n = 67) (Table 5).

**Table 3:** Distribution, Mean, and Median of the Length of Stay (Days), by Comorbidities (N = 157)

Comorbidity	Percent (n)	Mean LOS (SD)	Median^a^ (IQR) LOS	Range LOS	*P* value^b^
High blood pressure (HTN)	66.2 (104)	5.6 (2.9)	5.0 (4.0,6.0)	2-19	0.727
Hyperlipidemia (HLD)	38.2 (60)	5.2 (2.7)	4.0 (3.0,6.0)	2-15	0.395
Diabetes mellitus	17.2 (27)	4.9 (2.2)	4.0 (3.0,6.0)	2-11	0.274
Thyroid disorders	7.0 (11)	5.1 (1.8)	5.0 (3.0,7.0)	3-8	1.000
Gastrointestinal diseases	7.6 (12)	6.0 (2.0)	5.5 (5.0,7.5)	3-10	0.355
Renal dysfunction	5.1 (8)	6.1 (2.0)	6.0 (5.0,7.0)	3-10	0.140
Thromboembolic event	22.3 (35)	6.1 (3.5)	5.0 (3.0,7.0)	1-19	0.232
Atrial fibrillation	13.4 (21)	6.2 (3.7)	6.0 (4.0.8.0)	1-19	0.142
Heart disease nonmyocardial infarction (MI)	26.8 (42)	6.0 (2.8)	5.0 (4.0,7.0)	2-15	0.092
Lung pathology	11.5 (18)	5.8 (3.4)	5.0 (4.0,6.0)	2-15	1.000
Cancer	15.3 (24)	5.8 (3.3)	5.0 (4.0,7.0)	2-19	0.357
Dementia	7.6 (12)	5.8 (2.1)	5.5 (4.0,7.5)	3-10	0.355
Other mental health condition	2.6 (4)	4.3 (1.5)	4.0 (3.0,5.5)	3-6	1.000

IQR = interquartile range, LOS = length of stay

a Median, Fisher exact test.

b Analyses were restricted to patients without missing values.

**Table 4:** Distribution, Mean, and Median of the Length of Stay (Days), by Admission and Surgery-Related Factors (N = 157)

Admission	Percent (n)	Mean LOS (SD)	Median^a^ (IQR) LOS	Range LOS	*P* value^b^
Admission date					
Sunday	10.2 (16)	5.6 (2.8)	4.5 (3.0,8.5)	3-10	0.342
Monday	10.8 (17)	4.0 (1.6)	4.0 (3.0,4.0)	1-7	
Tuesday	17.8 (28)	5.0 (2.1)	4.5 (3.0,6.5)	3-10	
Wednesday	21.0 (33)	5.9 (2.4)	6.0 (5.0,7.0)	2-13	
Thursday	13.4 (21)	5.4 (1.9)	5.0 (4.0,6.0)	2-11	
Friday	12.1 (19)	5.6 (2.8)	5.0 (4.0,6.0)	3-15	
Saturday	14.7 (23)	5.6 (4.4)	4.0 (3.0,5.0)	2-19	
Admission ward					
Surgical	83.4 (131)	5.2 (2.6)	5.0 (3.0,6.0)	1-15	0.142
Telemetry	5.7 (9)	5.8 (1.7)	5.0 (5.0,6.0)	4-9	
PCU	6.4 (10)	7.6 (4.5)	6.0 (5.0,8.0)	3-19	
Medical	3.2 (5)	4.4 (2.1)	4.0 (3.0,4.0)	3-8	
Intensive care unit (omitted)^c^	1.3 (2)				
ASA score					
ASA II	14.0 (22)	4.2 (1.8)	4.0 (3.0,6.0)	2-8	0.570
ASA III	66.9 (105)	5.6 (3.0)	5.0 (4.0,6.0)	1-19	
ASA IV	18.5 (29)	5.7 (2.1)	5.0 (4.0,7.0)	3-10	
ASA V (omitted)^c^	0.6 (1)				
Days from admission to surgery					
Less than one day	21.7 (34)	4.1 (1.9)	4.0 (3.0,5.0)	1-9	0.001
One to two days	73.3 (115)	5.6 (2.8)	5.0 (4.0,6.0)	2-19	
More than two days	5.1 (8)	7.4 (2.3)	7.0 (6.0,9.0)	4-11	

IQR = interquartile range, LOS = length of stay

a Median, Fisher exact test.

b Analyses were restricted to patients without missing values.

c Omitted information, less than 5 cases.

**Table 5:** Distribution, Mean, and Median of the Length of Stay (Days), by Admission and Surgery-Related Factors (N = 157)

Surgery Factor	Percent (n)	Mean LOS (SD)	Median^a^ (IQR) LOS	Range LOS	*P* value^b^
Type of surgery					
Hemiarthroplasty	57.3 (90)	5.0 (2.4)	4.5 (3.0,6.0)	2-13	0.133
Cephalomedullary device fixation	42.7 (67)	5.9 (3.1)	5.0 (4.0,7.0)	1-19	
Admission team					
Orthopaedic surgery	42.0 (66)	5.1 (2.7)	4.0 (3.0,6.0)	2-15	0.053
General surgery	31.2 (49)	5.7 (2.8)	5.0 (4.0,7.0)	2-19	
Medicine	26.8 (42)	5.3 (2.5)	5.0 (4.0,6.0)	1-13	
Surgeon					
Surgeon A	29.9 (47)	5.4 (2.2)	5.0 (4.0,6.0)	3-6	0.921
Surgeon B	7.0 (11)	5.9 (4.9)	5.0 (3.0,6.0)	2-19	
Surgeon C	29.3 (46)	5.3 (2.5)	5.0 (4.0,6.0)	2-13	
Surgeon D	33.8 (53)	5.3 (2.7)	4.0. (3.0,7.0)	1-15	
Discharged status					
Skilled nursing facility	53.5 (84)	5.6 (2.9)	5.0 (4.0,6.5)	1-19	0.317
Inpatient rehabilitation	42.7 (67)	5.1 (2.5)	4.0 (4.0,6.0)	2-15	
Home with home care	3.8 (6)	5.0 (2.4)	5.0 (3.0.7.0)	2-8	

IQR = interquartile range, LOS = length of stay

^a^Median, Fisher exact test.

^b^Analyses were restricted to patients without missing values.

### Bivariate Analyses

The first five tables present the results of the bivariate analyses; the *P* values indicate if the LOS medians are statistically different between groups (eg, female versus male). The results of bivariate analyses suggest that patients with preoperative anemia had a longer median LOS than those without preoperative anemia (5 days versus 4 days, respectively; *P* = 0.029); patients with an in-hospital blood transfusion (BT) had a longer median LOS than patients without BT (6 days versus 4 days respectively; *P* = 0.022); and the group of patients who had the hip fracture surgery after 2 or more days of hospital admission had a significantly longer median LOS than their counterparts (7 days versus 4 days, respectively; *P* = 0.022).

### Adjusted Regression

The best parsimonious final TNBR model included the following factors: age, sex, days from admission to surgery, living arrangements, smoking status and nutritional status, sepsis, and presence of a thromboembolic event. After controlling for the other factors in the regression, older patients were more likely to have longer LOS (*P* = 0.016); patients with surgery one or two days (*P* = 0.007) or more than two days (*P* = 0.001) after admission date had a longer LOS than those who had it within the first 24 hours; patients who live in institutions had a shorter LOS than those that live in their own home or with family members (*P* = 0.034); current smokers had a longer LOS than never users (*P* = 0.008); malnourished patients had a longer LOS than nonmalnourished patients (*P* = 0.003); patients with sepsis had a longer LOS than their counterparts (*P* = 0.041); and patients with a thromboembolic event as part of their medical history had a higher LOS than those without it (*P* = 0.034) (Table 6).

**Table 6:** Factors Associated With Length of Stay and Results of the Truncated Negative Binomial Regression of LOS as the Main Outcome (N = 145)

Characteristic	IRR	St Err	LL 95% CI	UL 95% CI	*P* value
Age	1.014	0.0058	1.002	1.025	0.016
Sex					
Female (reference)	—	—	—	—	
Male	1.194	0.1125	0.993	1.436	0.059
Smoking status					
Never (reference)	—	—	—	—	
Former	0.974	0.0753 0.1968	0.837	1.133	0.730
Current	1.439		1.101	1.882	0.008
Living arrangements					
Home or living with family (reference)	—	—	—	—	
Other living arrangements^a^	0.792	0.0872	0.638	0.983	0.034
Days from admission to surgery					
Less than one day (reference)	—	—	—	—	
One to 2 days	1.273	0.1146	1.067	1.518	0.007
More than 2 days	1.564	0.2163	1.192	2.051	0.001
Nutritional status					
Nonmalnourished (reference)	—	—	—	—	
Malnourished	1.231	0.0875	1.071	1.415	0.003
Concurrent sepsis—SIRS criteria					
No (reference)	—	—	—	—	
Yes	1.247	0.1345	1.009	1.541	0.041
Thromboembolic event					
No (reference)	—	—	—	—	
Yes	1.249	0.1316	1.017	1.536	0.034

IRR = incidence rate ratio, St Err = standard error, LL 95% CI = lower level 95% confidence interval, UL 95% CI = upper level 95% confidence interval

^a^Other living arrangements included nursing homes, group homes, or assisted living.

## Discussion

The main findings of this study may be summarized succinctly. First, the mean LOS in our study was 5.36 days (1 to 19 days) with a median of 5 days (IQR 4 to 6 days). Second, the bivariate test results suggested that preoperative anemia, in-hospital blood transfusion, and days from admission to surgery are factors statistically significantly associated with a longer median LOS. Third, the adjusted regression model suggested that in this study sample, the factors associated with a longer LOS were age (older patients), days from hospital admission to surgery, smoking status, nutritional status (malnourished), sepsis, and thromboembolic events. The results supported our hypothesis as we found preoperative anemia, receiving transfusions, smoking, body mass index (BMI), delay in surgery from time of admission, and coming from home or living with family were factors that increased the LOS.

To our knowledge, this is the first study looking specifically at the factors associated with LOS of geriatric hip fractures in a community hospital setting. Previous studies have examined hip fractures admitted to trauma centers or examined national healthcare databases.^9,10,11,12,13^ Their findings have been helpful in the evaluation of processes and procedures at larger tertiary care centers and have been informative in establishing clinical operating guidelines, including the use of dedicated orthogeriatric comanagement.^12,14^ However, these resources are not always readily available at smaller community-based hospitals.

Studies have shown a link between longer postoperative inpatient LOS with higher postsurgical mortality after hip fracture surgeries in the geriatric population.^1,2^ However, there have been a limited number of publications focused on describing risk factors that may increase hospitalization LOS in this population. Notably, in 2019, Hecht, et. al^10^ published a report attempting to construct a predictive model for geriatric hip fracture patients. They found that reduced time to surgery and absence of delirium were predictors of shorter LOS.^10^ In addition, they reported an average LOS of patients undergoing hip fracture surgical repair of 5.95 days in their retrospective trial at the UC Davis Geriatric Fracture Program. The findings of Hecht and colleagues are in concordance with the result of our community hospital settings, where the average LOS was 5.36 days, and where reduced time to surgery was associated with a lower median LOS.^10^

Another factor associated with longer median LOS in our study sample was preoperative anemia (*P* = 0.029). Our results showed that patients with preoperative anemia had an average LOS of 5.9 (SD = 2.7) days versus patients without anemia LOS of 4.7 (SD = 2.5) days. The reason for this is probably multifactorial, but this trend has been seen in the literature. Preoperative anemia is a factor for early morbidity/mortality.^9^ In a 2020 study by Sim et al,^15^ it was found that geriatric patients with preoperative anemia and sustaining hip fracture had lower functional outcomes after surgery than those without anemia. A common theme in both studies was the association between preoperative anemia and comorbid conditions. Patients with preoperative anemia were shown to have more comorbidities, and this was postulated to be the driving force behind their frailty after surgery.

An added risk of having a preoperative anemia is needing a perioperative transfusion, which can also lead to an increased LOS. Our results of bivariate analyses showed a significantly higher median LOS (*P* = 0.022) for patients receiving blood transfusions. This is consistent with Arshi et al^16^ and Leuzinger et al^17^ both separately reporting an association between blood transfusions and other postoperative complications, including total hospital LOS. Previous studies have found that patients with blood transfusion had lower hemoglobin levels on admission.^18^ Similarly, in our study sample, 93.55% of the patients with blood transfusion (29/31; *P* < 0.001) had preoperative anemia. Although in our bivariate analysis, preoperative anemia and blood transfusion were statistically significantly associated with longer LOS median, when adding them into the multivariate analyses, they were not significant anymore. It can be inferred that other factors in our model might have a moderation effect or that we did not have enough cases for power analyses. Further studies are needed to test possible associations in community hospital settings.

The results of our multivariate analyses suggested that the LOS for those living at home/family settings was statistically significantly longer in comparison to those coming from other living arrangements (nursing homes, group homes, or assisting living facilities). Most of our patients live at home or a family member's home (85.3%) with an average LOS of 5.4 days (range 2 to 19 days) compared with the patients who came from other facilities (14.7%) whose average LOS was 5 days (range 1 to 10 days). Consistent with our findings, a study focused on veterans and war widows hospitalized for hip fracture in Australia reported that patients from other facilities had a shorter LOS by roughly 6 days in comparison to those coming from home/family.^19^ Similarly, a study with older adults (≥65 years) admitted to a Canadian health region hospital showed that patients coming from a long-term care facility had LOS that was 4 days shorter than those coming from a home or with family.^20^ This is likely because patients coming from a facility already have a bed and are not required to undergo the authorization process typically required by insurance carriers to obtain a bed at the facility for continued rehab/care.

In our study sample, patients discharged to IPR facilities had an average and median LOS shorter than those discharged to skilled nursing facilities (SNFs); however, the difference was not statistically significant. This is partly attributed to having an IPR facility directly at our hospital, which allows for quicker transition for those that qualify. In addition, on admission, we routinely consult our Physical Medicine and Rehabilitation service as well as case management, social work, and physical therapy. Authorization for SNF and IPR facilities requires the patient to be in the hospital for three days not including the discharge date per the Code of Federal Regulations.^21^ We discussed this with our case management team to learn more about the process from their point of view. They informed us that based on the hospital payor contract established at our facility, insurance companies have 72 hours to respond to the request for SNF or IPR requests. Requests are sent once there is a verified medical need for rehab, and responses are usually obtained 48 hours after a request was made. In addition, case management at our facility mentioned that it generally takes longer for IPR approval due to insurance requests for peer to peer; however, our results do not reflect this delay.

The results of our bivariate and multivariate model showed that patients who have their operation completed less than one day of admission have significantly reduced LOS than their counterparts. Our physicians do not have a dedicated trauma OR room; however, they do have blocked time for their elective cases. As such, our physicians typically take call the night before they have block time scheduled, so patients with hip fracture can be added to their preexisting block time. If these patients are not able to be added on to their block time, we will conduct the case as an add-on during lunch in their clinic day or after clinic is done. This is done to accommodate the patient having surgery <48 hours after arrival to the hospital. Consistent with our findings, an older adult hip fracture comanagement pathway study at an academic level I trauma center in California found that time to surgery and postoperative delirium were the most important factors predicting the patient's LOS.^10^ Although we did not evaluate for delirium in our patient set, we agree that increased time to surgery will increase the LOS.

Preoperative nutritional status was evaluated using albumin. We used a cutoff value of less than 3.4 mg/dL to be considered malnourished. Among our patient population, malnourished patients had a longer LOS than nonmalnourished patients. The most likely explanation for this is that malnourished patients probably had more postoperative complications than patients with normal albumin. It is known that low albumin can predict postoperative complications.^3,22^ In the elective orthopaedic population, these patients tend to have more surgical site infections.^22^ Also, in 2018, Ryan et al found that geriatric hip fracture patients with low albumin have a higher likelihood of death after hip fracture surgery.^23^

Patients we defined as having thromboembolic events in their medical history (eg, MI, stroke, DVT, and PE) had a higher LOS than their counterparts. The most likely reason for this was preoperative medical optimization for blood thinners and a cardiology consult. Gautier et al^24^ showed in a 2020 retrospective study that patients on direct oral anticoagulants (DOACs) were more likely to experience surgical delay. Of note, however, they found that the surgical delay was not >48 hours, which is in concordance with current recommendations on treatment of hip fractures. Belotti et al. found in a 2013 study that patients on antiplatelet therapy were at an increased risk of delaying surgery.^25^ There is still controversy over the benefits and risks of delaying surgery in patients taking anticoagulants or antiplatelets. However, recent studies are advocating for early surgery in patients taking DOACs or antiplatelet therapy.^26,27^ Indeed, the 2020 Association of Anesthetist's guideline on the management of hip fractures recognizes risks of delay and does not recommend delaying surgery based on taking an antiplatelet agent.^12^ However, they do recommend that a surgical delay of two half-lives of a DOAC (∼24 hours after the last dose) balance the risk and benefit.

The American College of Cardiology and the American Heart Association have made guidelines to help elucidate who needs a preoperative cardiology consultation because of increased cardiovascular risk.^28^ However, it has been shown in a 2020 study by Hoehmann et al that these guidelines are often not followed. Their retrospective review at a Level 1 trauma center and their affiliated safety net hospital showed that 18% of patients met the criteria for preoperative cardiology consultation according to the guidelines; however, 44% of patients received one.^29^ They also showed that 34% of patients met the criteria to receive a preoperative echocardiogram, but almost 90% of patients received one.^29^ This resulted in a significant delay in surgery.^29^ Our study did not specifically look to see if cardiology consults and procedures caused any delay, but we did note that if surgery occurred on the second day or later, there was a statistically significant increase in LOS.

Smoking tobacco seems to affect every part of orthopaedics. Geriatric hip fractures are no different as we found a significant increase in the LOS median for active smokers after controlling for other covariates. Smoking leads to greater wound complications and a longer time to fracture union.^30^ Another reason for the increased LOS is likely attributed to smokers having even more severe medical comorbidities than the average geriatric patient. An interesting animal study conducted by Hao et al^31^ in 2013 showed a greater increase in inflammatory markers and greater lung injury in animals with a hip fracture that were exposed to chronic cigarette smoke compared with those that were not.

There were a few factors that interestingly did not reach statistical significance. For example, we were expecting admission service, ASA score, and the day of the week admitted to affect the LOS. Many studies had improved outcomes for comanagement of hip fracture patients between orthopaedics and internal medicine/geriatrics, and it has been included in the American Academy of Orthopaedic Surgeons Clinical Guideline regarding the treatment of hip fractures.^12,14^ At our institution, we use an integrated care model for all our hip fracture patients where no matter which service admits the patient, each team has an active role in the management of the patient. This helps expedite medical management before surgery, time to surgery, and disposition out of the hospital. We believe that this is the reason we observed no difference in LOS based on the clinical team the patient was admitted to. ASA is a score based on comorbidities designated by anesthesia.^8^ Studies have often found an association with increased ASA score and increased LOS.^32^ Our research did not show this, and we can only conclude that this is due to not having enough patients in the study. In general, we agree that a higher ASA would be a risk factor for an increased LOS.

In terms of days of the week, contrary to previous study findings, we did not find differences in LOS. In 2021, Rashidifard et al conducted a retrospective review of 100 hip fractures over 1 year at a Level 1 trauma center.^33^ They found a longer LOS associated with patients who were admitted on Thursdays.^33^ They postulated that this was because of decreased hospital staffing on the weekend related to physical therapy and case management.^33^ Although we did not find a difference in our data, we do feel that weekend staffing does play a role in patient LOS.

The study limitations merit attention. First, in terms of our methods, we did not analyze the difference in LOS by the rate of postoperative complications. This limits our ability to draw any specific conclusions about the rate of complications associated with increased LOS. Additional limitations to our study would be that we may not have had enough power to find a significant difference in some variables.

Future research could be conducted looking into the same factors regarding LOS but separating the different types of fractures. This would allow for the ability to have appropriate expectations of LOS based on the fracture type and numerous variabilities available. If done with sufficient patient populations, this could provide significant data to the healthcare industry at large to provide appropriate expectations to patients, family, providers, and insurance companies. These data would help those understand the factors that play into the patient's care, reasons for delays in discharge, and discharge locations.

## References

[1] Brauer CA Coca-Perraillon M Cutler DM Rosen AB: Incidence and mortality of hip fractures in the United States. JAMA 2009;302:1573-1579. 19826027 10.1001/jama.2009.1462 PMC4410861

[2] Bohl DD Shen MR Hannon CP Fillingham YA Darrith B Della Valle CJ: Serum albumin predicts survival and postoperative course following surgery for geriatric hip fracture. J Bone Joint Surg 2017;99:2110-2118. 29257017 10.2106/JBJS.16.01620

[3] Cross MB Yi PH Thomas CF Garcia J Della Valle CJ: Evaluation of malnutrition in orthopaedic surgery. J Am Acad Orthop Surg 2014;22:193-199. 24603829 10.5435/JAAOS-22-03-193

[4] Roberts KC Brox WT Jevsevar DS Sevarino K: Management of hip fractures in the elderly. J Am Acad Orthop Surg 2015;23:131-137. 25624365 10.5435/JAAOS-D-14-00432

[5] Means RT: Approach to the anemias, in Goldman L Schafer AI, eds, ed 26. Philadelphia, PA, Elsevier, 2020.Goldman-Cecil Medicine.

[6] World Health Organization: Physical status: The use and interpretation of anthropometry: Report of a World health organization (WHO) expert committee. World Health Organ Tech Rep Ser 1995;845:1-452. 8594834

[7] Bone RC Balk RA Cerra FB : Definitions for sepsis and organ failure and guidelines for the use of innovative therapies in sepsis. Chest 1992;101:1644-1655. 1303622 10.1378/chest.101.6.1644

[8] Mayhew D Mendonca V Murthy BVS: A review of ASA physical status - historical perspectives and modern developments. Anaesthesia 2019;74:373-379. 30648259 10.1111/anae.14569

[9] Ryan G Nowak L Melo L : Anemia at presentation predicts acute mortality and need for readmission following geriatric hip fracture. JBJS Open Access 2020;5:e20.00048. 10.2106/JBJS.OA.20.00048 PMC7722583 33299961

[10] Hecht G Slee CA Goodell PB Taylor SL Wolinsky PR: Predictive modeling for geriatric hip fracture patients: Early surgery and delirium have the largest influence on length of stay. J Am Acad Orthop Surg 2019;27:e293-e300. 30358636 10.5435/JAAOS-D-17-00447 PMC6411423

[11] Prestmo A Hagen G Sletvold O : Comprehensive geriatric care for patients with hip fractures: A prospective, randomised, controlled trial. The Lancet 2015;385:1623-1633. 10.1016/S0140-6736(14)62409-0 25662415

[12] Patel JN Klein DS Sreekumar S Liporace FA Yoon RS: Outcomes in multidisciplinary team-based approach in geriatric hip fracture care: A systematic review. J Am Acad Orthop Surg 2020;28:128-133. 31977613 10.5435/JAAOS-D-18-00425

[13] Ricci WM Brandt A McAndrew C Gardner MJ: Factors affecting delay to surgery and length of stay for patients with hip fracture. J Orthop Trauma 2015;29:e109-e114. 25186844 10.1097/BOT.0000000000000221 PMC4339640

[14] Baroni M Serra R Boccardi V : The orthogeriatric comanagement improves clinical outcomes of hip fracture in older adults. Osteoporos Int 2019;30:907-916. 30715561 10.1007/s00198-019-04858-2

[15] Sim YE Sim SeD Seng C Howe TS Koh SB Abdullah HR: Preoperative anemia, functional outcomes, and quality of life after hip fracture surgery. J Am Geriatr Soc 2018;66:1524-1531. 30091139 10.1111/jgs.15428

[16] Arshi A Lai WC Iglesias BC : Blood transfusion rates and predictors following geriatric hip fracture surgery. HIP Int 2020;31:272-279. 31912747 10.1177/1120700019897878

[17] Leuzinger E Poblete B Konrad CJ Hansen D: How current transfusion practices in geriatric patients with hip fracture still differ from current guidelines and the effects on outcome. Eur J Anaesthesiology 2018;35:972-979. 10.1097/EJA.0000000000000883 30234668

[18] Adunsky A Lichtenstein A Mizrahi E Arad M Heim M: Blood transfusion requirements in elderly hip fracture patients. Arch Gerontol Geriatr 2003;36:75-81. 12849101 10.1016/s0167-4943(02)00059-6

[19] Ireland AW Kelly PJ Cumming RG: Total hospital stay for hip fracture: Measuring the variations due to pre-fracture residence, rehabilitation, complications and comorbidities. BMC Health Serv Res 2015;15:17. 25609030 10.1186/s12913-015-0697-3 PMC4308914

[20] Beaupre LA Cintas JG Jones CA : Does functional recovery in elderly hip fracture patients differ between patients admitted from long-term care and the community?. J Gerontol A Biol Sci Med Sci 2007;62:1127-1133. 17921426 10.1093/gerona/62.10.1127

[21] 42 C.F.R. § 409.30.

[22] Simons MJ Amin NH Scuderi GR: Acute wound complications after total knee arthroplasty: Prevention and management. J Am Acad Orthop Surg 2017;25:547-555. 28737615 10.5435/JAAOS-D-15-00402

[23] Ryan S Politzer C Fletcher A Bolognesi M Seyler T: Preoperative hypoalbuminemia predicts poor short-term outcomes for hip fracture surgery. Orthopedics 2018;41:e789-e796. 30222797 10.3928/01477447-20180912-03

[24] Gautier N Pirson A Lechat JP Van Der Linden P: Impact of direct oral anticoagulant therapy on operative delay, blood loss, transfusion and postoperative morbidity mortality in hip fracture patient, an observational study. Thromb Res 2020;194:165-167. 32788110 10.1016/j.thromres.2020.06.021

[25] Belotti LMB Bartoli S Trombetti S Montella MT Toni A De Palma R: Factors influencing surgical delay after hip fracture in hospitals of emilia romagna region, Italy: A multilevel analysis. HIP Int 2013;23:15-21. 23397198 10.5301/HIP.2013.10717

[26] Lott A Haglin J Belayneh R Konda SR Leucht P Egol KA: Surgical delay is not warranted for patients with hip fractures receiving non-warfarin anticoagulants. Orthopedics 2019;42:e331-e335. 30913296 10.3928/01477447-20190321-02

[27] Schermann H Gurel R Gold A : Safety of urgent hip fracture surgery protocol under influence of direct oral anticoagulation medications. Injury 2019;50:398-402. 30391072 10.1016/j.injury.2018.10.033

[28] Fleisher LA Fleischmann KE Auerbach AD : 2014 ACC/AHA guideline on perioperative cardiovascular evaluation and management of patients undergoing noncardiac surgery: A report of the American College of cardiology/American heart association task force on practice guidelines. Circulation 2015;130:e278-e333. 10.1161/CIR.0000000000000106 25085961

[29] Hoehmann CL Thompson J Long M : Unnecessary pre-operative cardiology evaluation and transthoracic echocardiogram delays time to surgery for geriatric hip fractures. J Orthop Trauma 2021;35:205-210. 33079839 10.1097/BOT.0000000000001941

[30] Argintar E Triantafillou K Delahay J Wiesel B: The musculoskeletal effects of perioperative smoking. J Am Acad Orthop Surg 2012;20:359-363. 22661565 10.5435/JAAOS-20-06-359

[31] Hao Z Tiansheng S Zhi L Jianzheng Z Xiaowei W Jia L: Hip fracture aggravates systemic inflammation and lung injury in aged chronic cigarette smoke exposed rats. J Orthop Res 2014;32:24-30. 24115247 10.1002/jor.22491

[32] Kay H Sathiyakumar V Yoneda Z : The effects of ASA physical status on length of stay and inpatient cost in the surgical treatment of isolated orthopaedic fractures. J Orthop Trauma 2014;28:e153-e159. 10.1097/01.bot.0000437568.84322.cd 24149446

[33] Rashidifard CH Bush CM Muccino PP DiPasquale TG: Implications of admission and surgical timing on hospital length of stay in patients with hip fractures. J Am Acad Orthop Surg 2021;29:e79-e84. 33394614 10.5435/JAAOS-D-19-00129

